# To quell obesity, who should regulate food marketing to children?

**DOI:** 10.1186/1744-8603-1-9

**Published:** 2005-07-14

**Authors:** Ben Kelly

**Affiliations:** 1 Public Health Advocacy Institute in Boston, MA, USA

## Abstract

The global hegemony of the United States in the production and marketing of food, while a marvel of economic success, has contributed to the epidemic of obesity that is particularly afflicting children. So far the U.S. government has declined to regulate the aggressive ways in which food producers market high-energy, low-nutrition foods to young people. That public-health responsibility has been left to an industry-created scheme of self-regulation that is deeply flawed; there is a compelling need for government involvement. The issue is certain to be raised by health advocates at a U.S. Federal Trade Commission meeting in mid-July to discuss the self-regulatory approach, but the outlook for remedies to emerge from the meeting is not encouraging.

## 

United States businesses have been unsurpassed leaders in proliferating the availability, marketing and consumption of high-energy, low-nutrition foods at home and around the world. Their aggressive, high-priced marketing of those foods has especially targeted children – the adult consumers of the future in whom the early creation of brand and product commitment is, in the eyes and ledgers of some huge corporations, essential to profitability.

But the leadership in so-called "junk food" promotion has not been matched in the U.S. by effective regulatory controls to prevent marketing abuses of children. Advertising of such products has been misleading, overblown, and seemingly bent on undermining the ability of parents to moderate their children's eating practices. Although still concentrated in television viewing hours directed at child entertainment and publications read by children, it is spreading rapidly to other means of messaging, among them online games ("advergames"), product placements in films and television shows, and product-linked websites.

The position of the driving-force industries – food marketers and advertising agencies – presents an interesting internal contradiction. On one hand they assert valid evidence is lacking that exposure to such messages actually influences the consumption practices of children, let alone contributes to health problems such as obesity. On the other they claim that abusive child-directed marketing is being effectively and adequately controlled by an industry-sponsored system of self-regulation. Health advocates, meanwhile, are increasingly urging that the government intervene by legislating and enforcing objective standards over and above any that the industry imposes or claims to impose.

The regulatory agency that would set and implement such standards is the U.S. Federal Trade Commission (FTC), but at present it contends it cannot do so without additional statutory authority. Some in Congress, prominent among them Senator Tom Harkin, have proposed legislation to create such authority, but Harkin and like-minded lawmakers are members of the minority Democratic Party. President Bush and the majority members of Congress are on record as opposing strictures on the scope of food marketing; the view was reflected in efforts by U.S. spokespersons last year, made on behalf of American food companies, to rein in the World Health Organization's proposed nutrition guidelines.

FTC now plans to hold a public workshop, "Perspectives on Marketing, Self-Regulation, and Childhood Obesity," in Washington, D.C. on July 14–15, jointly with the Department of Health and Human Services. The description of the workshop – an "open discussion of self-regulation and the marketing of food and beverages to children" – suggests that the option of government controls will not be considered.

If that is the case, the proceeding will be limited to discussions of the adequacy of the existing self-regulatory scheme. The locus of that scheme, operated by the food and advertising industries, is the 20-year-old Children's Advertising Review Unit (CARU). It comprises a five-person staff within the Council of Better Business Bureaus and is linked organizationally to a network of trade groups representing manufacturers and advertising agencies. Documents found on CARU's website, , describe the history, governance and mission of the activity. They do so with scant reference to public-health needs.

CARU claims for itself a high level of effectiveness, but does so by using measures that are seriously flawed. The measures assume that the guidelines and principles that CARU claims to impose on child-directed food marketing messages are meaningful. They imply that CARU in fact proactively sees, screens, and acts on a large body of such messages. And they suggest that CARU has powers to enforce its decisions against messages found to violate its guidelines and principles. In fact, none of these assumptions is accurate.

A recent article in the Wall Street Journal, the leading pro-business newspaper in the United States, put into perspective the reality of CARU's relationship to the marketing activities it claims to regulate. Under the headline "General Mills Touts Sugary Cereal as Healthy Kids Breakfast," the June 22 article reported that the giant food packager "plans to launch a national ad campaign targeted at children that will tout the health benefits of eating breakfast cereal – including Trix, Cocoa Puffs and other sugary ones it sells."

After noting the controversial nature of the General Mills plan and the opposition it is provoking among obesity control proponents, the article noted that CARU "is endorsing the General Mills campaign after the company sought the organization's input. 'I think it is responsible advertising,' said Elizabeth Lascoutx, director of the group, which is partly funded by children's advertisers, including General Mills. 'They're encouraging a behavior that is healthful' as opposed to not eating breakfast." Subsequently Lascoutx was quoted – in a news story originating in Golden Valley, Minnesota, General Mills's home base – as saying, "This is exactly what a leader in the food industry should be doing. Ensuring that positive, nonbranded health messages like 'Choose Breakfast' are being delivered to children is not only responsible, but commendable."

CARU's level of comfort with the pitching of sugared cereals to kids in the midst of an obesity epidemic is consistent with the intimacy of its connections with the very companies whose activities it is supposed to regulate. For instance, its funding comes from such industry giants, to name a few, as Burger King Corp., Frito-Lay, Inc., Grocery Manufacturers of America, Inc., Hershey Foods Corp., Kellogg Company, Kraft Foods, Inc., Masterfoods USA, McDonald's Corporation, National Confectioners Association, Nestle USA, Inc., Pepsico Beverages & Food, Sara Lee Corporation, and – last but clearly not least – General Mills itself.

At its July public meeting FTC is sure to hear from health advocates about the child-targeting marketing behavior of General Mills and other volume purveyors of high-energy, low-nutrition food to children. Some of those advocates will be urging the agency to use its existing authority to regulate that behavior, and to seek additional statutory authority if it is needed. It is likely, though, that the agency will turn a deaf ear to such entreaties and instead will emphasize the alleged benefits of the CARU scheme while asking for suggestions to strengthen it.

In a paper submitted to the FTC meeting docket, the Public Health Advocacy Institute , a Boston-based organization working to foster greater support for public health goals by the law community, has sharply disputed the premise that the CARU scheme works, or can be made to work, effectively. "Industry Controls Over Food Marketing: Are They Effective?" reviews a range of global assessments of self-regulation in general. It presents an extensive review and summary of leading commentaries addressing the world-wide state of regulation directed at food advertising that targets children. It concludes that the U.S. self-regulatory system is ineffective when measured against available criteria for gauging the adequacy of self-regulation, and also ineffective in the context of the worsening obesity epidemic and its damaging impact on children.

Using benchmarks drawn from a number of studies and commentaries concerned with self-regulation that have been published in the U.S. and abroad, the PHAI paper finds the CARU scheme and its potential for effectiveness to be seriously flawed and not remediable. It notes among other things that CARU fails to "provide an adequate public interest response" to public health needs; lacks "strong independent input, well-resourced monitoring and tough sanctions for breaches of the rules"; applies subjective criteria in assessing advertisements; does not review advertising prior to dissemination; lacks third-party review of its decision, and cannot enforce its decisions, which can be ignored by advertisers

Further, according to the paper, CARU fails to meet self-regulatory criteria indicated for the global business community (of which leading food marketers are a major component) in a study published by the United Nations Research Institute for Social Development. ^1 ^Its failures include:

• Lack of independent monitoring, which is "crucial" to effective self-regulation. "...implementation can only be guaranteed where there is an element of independent monitoring of codes of conduct."

• Unrealistic performance claims, which have sometimes "led to a worsening of the situation of those whom they purport to benefit."

• Adoption of self-regulatory schemes "simply to pre-empt external pressure."

• Weaknesses in implementation and compliance, such as a lack of "clear methods of implementation and means to ensure that it is being complied with..."

• Discouragement of stakeholder involvement: "It is in this area that the contrast between rhetoric and reality is particularly jarring. In the absence of independent monitoring and verification, it is difficult to evaluate whether company codes are applied extensively in practice or remain mere expressions of good intentions."

• Absence of sanctions.

How will these conclusions influence the FTC at its July public meeting? Although the outlook for health advocates is not encouraging given the agency's long-avowed support for CARU's self-regulatory scheme and the reflexive aversion to regulation of the party in power in Washington, that has not deterred groups such as Campaign for a Commercial-Free Childhood, The Center for Informed Food Choices, and the Strategic Alliance for Healthy Food and Activity Environments from raising the issue in their comments to the agency.

In contrast, the Grocery Manufacturers Association, speaking for the most powerful food corporations in America, has made clear that it expects the agency to be fully supportive of the CARU self-regulation approach. The issues it proposes to see raised at the July meeting emphasize "the role of self-regulation in promoting responsible advertising, including to children," and "how advertising will be part of the solution to the problem of obesity." If nothing else comes of the FTC meeting, it will at least be worth watching to see whether the Association will be challenged to reconcile its notion of advertising as "part of the solution to the obesity problem" with the new push to sell sweetened cereals being launched by one of its biggest members, General Mills.

## Note

^1^Rhys Jenkins, *Corporate Codes of Conduct Self-Regulation in a Global Economy*, Technology, Business and Society. Programme Paper Number 2, April 2001

